# Expanding the horizons of endoscopic ultrasound: diagnosis of non-digestive pathologies

**DOI:** 10.1093/gastro/got033

**Published:** 2014-01-08

**Authors:** Georgios Mavrogenis, Hocine Hassaini, Alain Sibille, Sofia Feloni, Pierre H. Deprez, Cédric Gillain, Philippe Warzée

**Affiliations:** ^1^Department of Gastroenterology, Grand Hôpital de Charleroi, Charleroi, Belgium and ^2^Department of Hepato-Gastroenterology, Cliniques universitaires Saint-Luc, Université Catholique de Louvain, Brussels, Belgium

**Keywords:** Endoscopic ultrasound, thyroid cyst, renal tumor, pulmonary embolism, aortic aneurysm, pneumonia, cardiac failure

## Abstract

Endoscopic ultrasound (EUS) is mainly used for the evaluation and sampling of mediastinal and abdominal lymph nodes, luminal and submucosal lesions of the upper and lower gastrointestinal tract, as well as in the diagnostic approach for pancreatic, biliary and liver disease. However, several non-digestive pathologies may be encountered as well, expanding the diagnostic potential of EUS. In this article, we present nine examples of extra-digestive abnormalities detected by means of EUS, including pathologies of the thyroid gland, mediastinal and abdominal vessels, lungs, kidney and the urinary bladder. The purpose of this article is to review the capabilities of EUS beyond routine evaluation of gastrointestinal organs.

## BACKGROUND

Endoscopic ultrasound (EUS) is a well-established imaging modality and plays an important role in the management of gastrointestinal and pancreato-biliary diseases. Since the development of EUS in the 1980s, dramatic changes have occurred and EUS has advanced its role, from an expensive toy for a few experts to an indispensable tool of a modern endoscopy unit.

A diagnostic EUS exam is supposed to answer specific questions, such as the presence or absence of biliary stones, the characterization of pancreatic lesions and the sampling of mediastinal or abdominal lymph nodes. However, a complete upper EUS exam should not be focused only on the target organ, but also include a systematic approach and inspection of the mediastinum, celiac axis, pancreas, biliary tract and liver. A meticulous examination may sometimes reveal abnormalities of the adjacent structures. For example, exploration of the mediastinum gives the opportunity to visualize the pulmonary artery and potentially, detect a pulmonary thrombosis. Positioning of the endoscope in the second portion of the duodenum permits the inspection of the right kidney and its vessels and, consequently, may lead to the detection of renal tumour. To further elaborate this concept and show the potential diagnostic implications of EUS, we present in this case series some examples of extra-digestive pathologies encountered during an EUS examination.

## METHODS

We conducted a retrospective review of our prospectively maintained image and video database of all EUS procedures performed between January 2011 and July 2013. The selection was restricted to cases with a clinically relevant endoscopic ultrasound observation concerning a non-digestive structure, excluding lesions of the adrenal glands. Among cases with similar findings, the case with the best or most representative images was chosen. A previously published case report from our institution is not included here [1].

All EUS exams were performed under general anaesthesia by four experienced endosonographers and one trainee, using a curvilinear array echoendoscope (GF-UCT180 Olympus, 5-10 MHz, Olympus, Belgium) and an Aloka Prosound a7 ultrasound processor (Aloka, Belgium). Images and videos were stored either to the internal memory of the ultrasound processor or to Telemis PACS storage service (Telemis, Belgium) via a Nebula image recorder (eSaturnus, Belgium). This study conforms with the principles outlined in the Declaration of Helsinki and was approved by the ethics review board of our hospital (Grand Hôpital de Charleroi, Charleroi, Belgium).

## CASE SERIES

### Case #1

**Figure 1.**
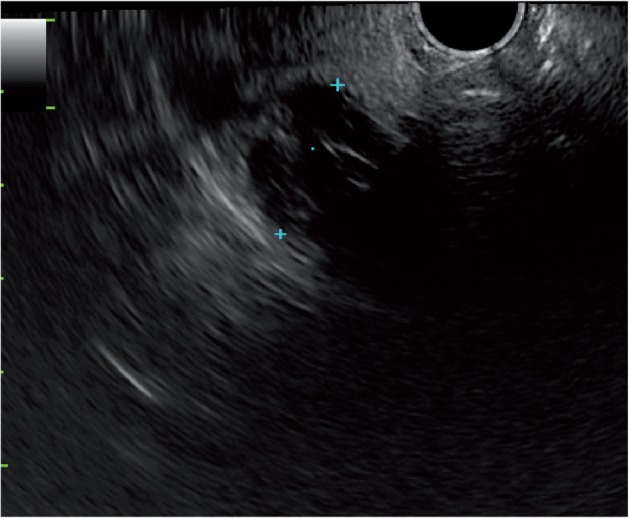
Incidental endoscopic ultrasound finding of a 17 mm multi-locular cystic lesion of the thyroid. The endoscope is advanced by 18–20 cm and is orientated towards the anterior wall of the oesophagus.

A 75-year-old woman underwent upper EUS for the investigation of recurrent epigastric pain. EUS revealed multiple biliary stones. In addition, examination of the thyroid gland disclosed numerous thyroid nodules as well as a 17 mm multilocular cyst (Figure 1). Percutaneous ultrasound confirmed the findings, blood tests demonstrated hypothyroidism and the patient was referred to an endocrinologist for further follow-up.

### Case #2

**Figure 2.**
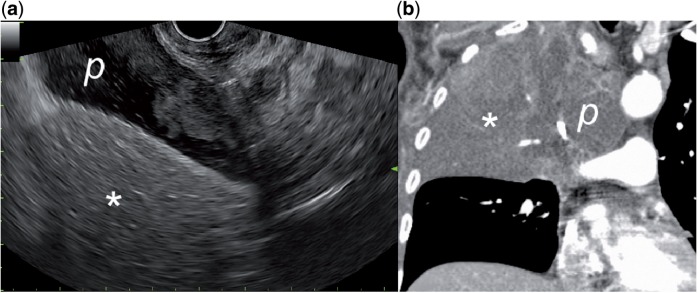
(a) Linear endoscopic ultrasound view of a large pleural effusion (p) surrounding an area with tissue-like pattern resembling that of liver (asterisk). The endoscope is positioned at the upper oesophagus and orientated towards the right lateral wall (clockwise direction). (b) Computer tomography image of the same anatomical region showing an atelectatic superior upper lobe surrounded by the pleural effusion.

A 57-year-old patient was admitted for fine needle aspiration (FNA) of a pancreatic mass. His previous medical history included a bronchial carcinoma of the right lung. EUS examination through the upper oesophagus disclosed a pleural effusion surrounding an area of tissue-like pattern corresponding to the upper superior lobe of the right lung (Figure 2). The diagnosis of lobar atelectasis was confirmed by a thoracic computer tomography (CT) scan.

### Case #3

**Figure 3.**
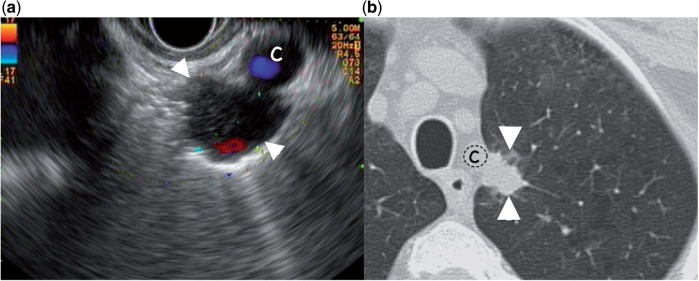
(a) Hypoechoic lesion of the superior left lobe (arrowheads) in close contact with the left subclavian artery (c). The endoscope is advanced at the level of the aortic arch and then rotated towards the left lateral wall of the oesophagus (counterclockwise direction). (b) Computer tomography image of the same lesion.

A 60-year-old patient with a history of chronic obstructive pulmonary disease was referred for FNA of a para-oesophageal pulmonary nodule of the superior left lobe (Figure 3). EUS disclosed the presence of multiple small mediastinal lymph nodes and a 15 mm hypoechoic pulmonary nodule in close contact to the left subclavian artery. FNA cytology showed necrosis without any signs of malignancy. Due to the suspicious character of the nodule, the

patient underwent an upper superior lobectomy. Polymerase chain reaction of the resection specimen revealed a Mycobacterium Xenopi infection and the patient was put on a triple antibiotic therapy.

### Case #4

**Figure 4.**
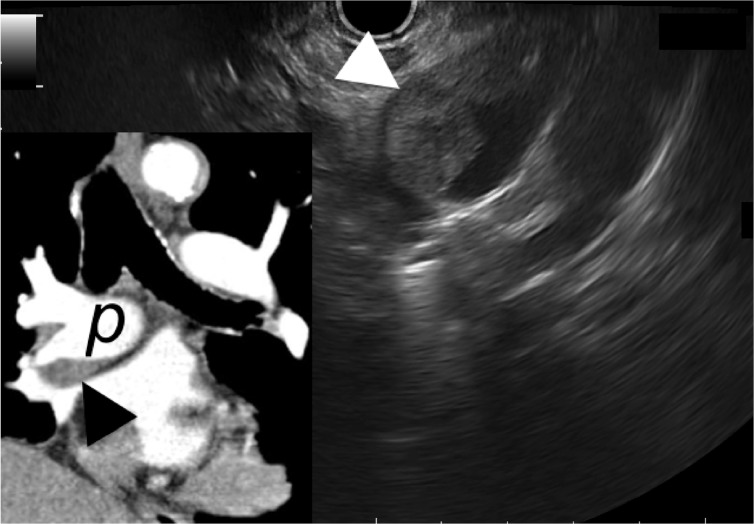
Endoscopic ultrasound image of a heterogeneous hypoechoic intraluminal lesion of the right pulmonary artery (arrowhead). The endoscope is advanced at the mid-oesophagus and orientated towards the anterior and right lateral wall of the oesophagus. Computer tomography scan showed an invasion of the right pulmonary artery (p) by the tumour (arrowhead).

A 75-year-old man with previous history of right-sided bronchial carcinoma was referred for the management of an acute non-alcoholic pancreatitis. An EUS exam was performed in order to rule out a biliary origin. Examination of the mediastinum disclosed an intraluminal hypoechoic lesion of the right pulmonary artery, suggesting a thrombosis (Figure 4). CT imaging was consistent with invasion of the right pulmonary artery by the bronchial cancer.

### Case #5

**Figure 5.**
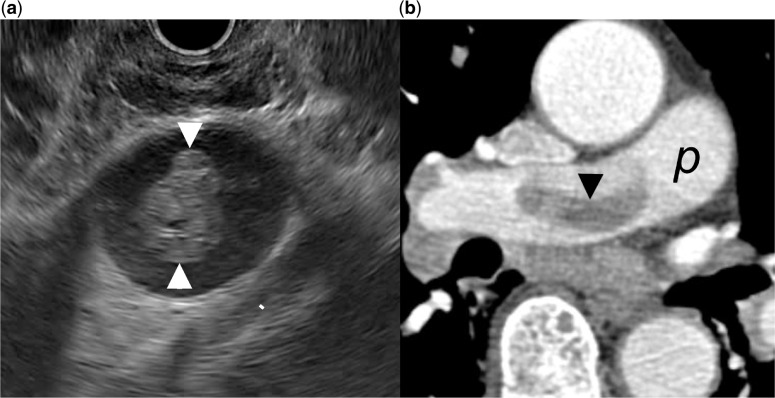
(a) Endoscopic ultrasound view of an intraluminal thrombus (arrowheads) of the main pulmonary artery. The endoscope is advanced at the mid-oesophagus and orientated towards the anterior wall of the oesophagus. (b) Computed tomography scan showed an extension of the thrombus to the right pulmonary artery (p).

An 85-year-old patient with mediastinal and abdominal adenopathy was referred for EUS-FNA. Surprisingly, the examination of the mediastinum revealed a mobile thrombus of the pulmonary artery (Figure 5). A subsequent CT scan confirmed our findings and showed a massive pulmonary embolism that extended to the left and right pulmonary artery.

### Case #6

**Figure 6.**
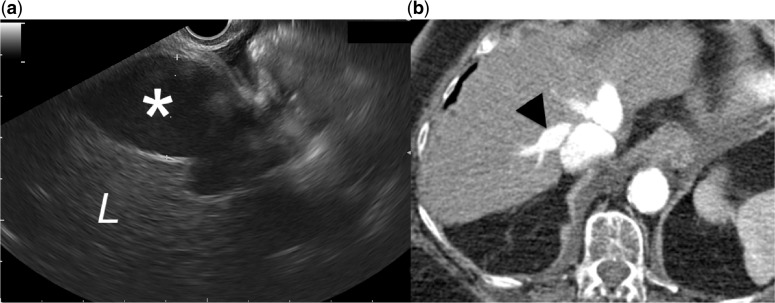
(a) Endoscopic ultrasound signs of right-sided heart failure: dilation of the hepatic veins and inferior vena cava (asterisk) to 25 mm. The endoscope is positioned 1–3 cm beyond the gastro-oesophageal junction and rotated clockwise. (b) Upper abdominal contrast-enhanced computer tomography section reveals retrograde opacification of the dilated hepatic veins (arrowhead), which is a specific sign of right-sided heart failure.

A 82-year-old woman presented with a non-viral cholestatic hepatitis. EUS exploration of the biliary tract and pancreas was normal. However, inspection of the liver showed dilated hepatic veins and inferior vena cava, with a biphasic waveform as well the presence of ascites. The combination of these findings indicated a right-sided heart failure and the patient was transferred to the cardiology department (Figure 6). This was later found to be secondary to severe pulmonary hypertension and concomitant tricuspid regurgitation.

### Case #7

**Figure 7.**
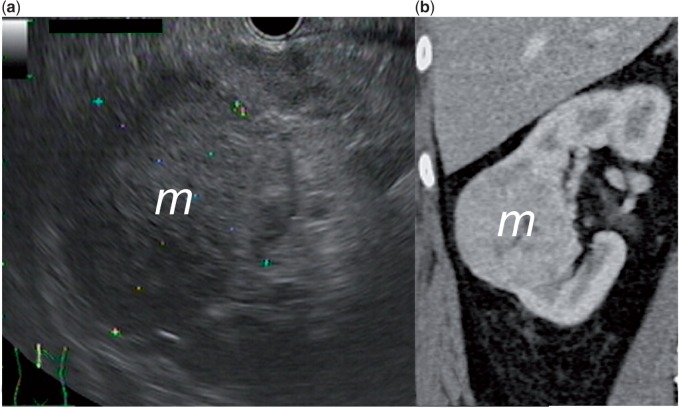
(a) Endoscopic ultrasound image of a 6 cm heterogeneous hypoechoic renal mass. (b) Computer tomography image of the same lesion.

A 62-year-old patient underwent EUS evaluation of a cystic lesion of the pancreas. Positioning of the endoscope to the second portion of the duodenum permitted the examination of the right kidney. This showed a 6 cm heterogeneous hypoechoic mass (Figure 7). The tumour was later resected and the histology showed a renal oncocytoma.

### Case #8

**Figure 8.**
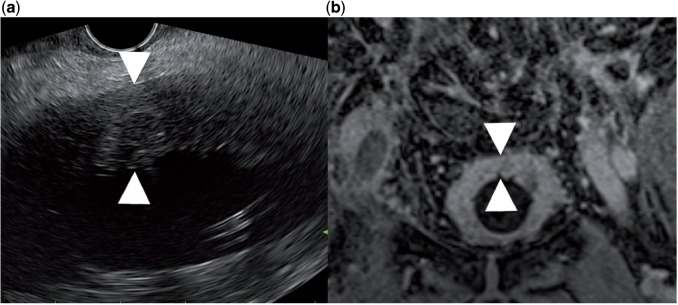
(a) Transrectal endoscopic ultrasound examination revealed an 11 mm thickening of the urinary bladder. The endoscope is orientated towards the anterior wall of the rectum. (b) This finding was confirmed by means of magnetic resonance imaging. Fine needle aspiration cytology disclosed a linitis-plastica carcinoma of the urinary bladder.

A 81-year-old patient was referred for EUS evaluation of wall thickening of the rectum. Inspection of the urinary bladder disclosed an 11 mm wall thickening (Figure 8). An FNA was performed showing a poorly differentiated urinary bladder carcinoma (linitis plastica) invading the rectum.

### Case #9

**Figure 9.**
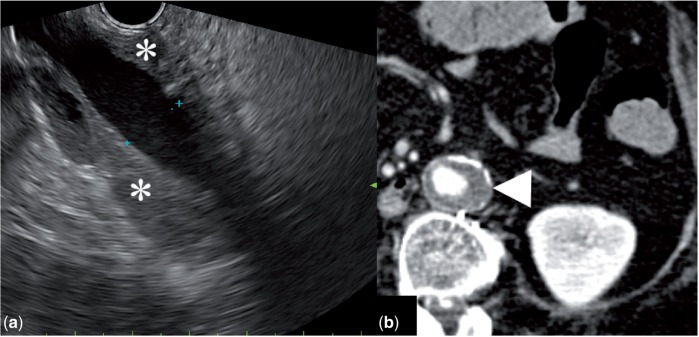
(a) Longitudinal EUS image of an infrarenal abdominal aortic aneurysm. The endoscope is positioned in the second portion of the duodenum, in a straight position and orientated towards the posterior-internal border of the duodenum. The asterisks highlight the circumferential thrombus that occludes approximately two thirds of the lumen. (b) Computer tomography image of the aneurysm.

A 62-year-old patient was admitted for EUS FNA of a 3 cm mediastinal lymph node. A complete upper EUS examination was performed. The endoscope was advanced to the distal duodenum for evaluation of the head of the pancreas. Inspection of the adjacent structures, revealed an infrarenal abdominal aortic aneurysm with a circumferential thrombus (Figure 9). CT scan confirmed the EUS findings.

## DISCUSSION

Ralph Waldo Emerson once said, “People only see what they are prepared to see …” This concept is crucial in the field of endoscopy, since endoscopic diagnosis of a particular pathology depends on the recognition of a specific image. A standard upper EUS exam is usually limited to the detection of mediastinal lymph nodes, to the inspection of the celiac axis, of the biliary tree and the liver, as well as of the pancreas. In addition, EUS is time-consuming and, with the increasing burden of examinations, endoscopists may become prone to limit their exam to the target area under investigation and avoid performing a complete examination. However, this attitude may result in failure to detect clinically relevant pathologies.

With this case series, we would like to emphasize two main points. First, that an EUS exam should not be limited on a single area but should also include a systematic and detailed examination. Secondly, we would like to draw the attention to the potential role of EUS in the diagnosis of ‘non-digestive’ pathologies. It may sound strange for an endoscopist to diagnose a pulmonary embolism or pneumonia; however, adding this information to an EUS exam report is clinically relevant, contributes to the global management of a clinical case and, finally, indicates a systematic and meticulous exam.

A good starting point for the diagnosis of extra-digestive pathologies is the identification of previously known lesions detected by means of other imaging modalities. In addition we suggest the following EUS stations: (i) positioning of the endoscope at the upper oesophagus gives the opportunity to inspect the thyroid gland which has the ultrasound aspect of an ‘ectopic pancreas’; (ii) identification of the pulmonary artery is easy and it could become part of a routine exam of the mediastinal lymph nodes; (iii) positioning of the endoscope close to the gastro-oesophageal junction and clockwise/counterclockwise rotation allows detection of small quantities of pleural fluid as well as of atelectasis; (iv) positioning at the gastric cardia or fundus and clockwise rotation permits the evaluation of the hepatic veins; (v) exploration of the tail of the pancreas gives the opportunity to evaluate the left kidney and adrenal gland, while positioning of the endoscope in the second portion of the duodenum allows inspection of the right kidney, adrenal gland and abdominal aorta. Progressively, endoscopists become familiar with non-digestive abnormalities, however, collaboration with an expert endosonographer or radiologist maybe necessary in order to avoid diagnostic pitfalls.

These concepts are further supported by several prior studies, in which, EUS was successfully used in the evaluation and diagnostic management of renal lesions [1–3], renal vein thrombosis [4], aortic intramural lesions [5], inferior vena cava thrombosis [4], pleural fluid collections [6] and pelvic diseases [7]. In particular, we would like to mention the expanding role of EUS-FNA in the management of lung masses located close to the oesophagus, since recent data suggest a 97% diagnostic accuracy, with a low complication rate of 1.6% [8].

In conclusion, with this article we would encourage endoscopic ultrasonographers to extend the horizons of EUS beyond the limits of current practice. Diagnosis of non-digestive pathologies during endoscopic ultrasound requires a methodical and careful approach to EUS examination but can yield important benefits for the patient.
